# Financial Capability and Economic Well-being in Later Life: A Population-based Study

**DOI:** 10.1093/geroni/igaf122.2333

**Published:** 2025-12-31

**Authors:** Yu-Chih Chen

**Affiliations:** National Taiwan University, Taipei, Taipei, Taiwan (Republic of China)

## Abstract

Financial capability, the interaction of financial literacy, access, and behavior, can influence individuals’ ability to manage finances and build economic stability over the life course. However, empirical evidence linking financial capability and economic well-being in old age is limited. We examine the components and mechanisms of the financial capability framework and investigate the differences between middle-aged (aged 50–64) and older adults (aged 65+) using nationally representative data in the United States. 12,840 individuals aged 50 and over were selected from the population-based 2018 National Financial Capability Study. Structural equation modeling and multigroup comparison were used to examine the mechanisms of financial capability on economic well-being and the moderating effects of age. Results showed that financial literacy and access were positively associated with financial behavior and subsequent economic well-being. The mediation results showed that financial literacy and access were equally important in contributing to economic well-being in later life. However, age had no moderating effect, suggesting these associations operated similarly in middle-aged and older adults. Findings suggest that strategies to promote economic well-being in later life through effective finance should be multifaceted, targeting each aspect of financial capability. Practices such as financial coaching and guidance, credit counseling, and financial profile assessments should be integrated into human and social services. Policies and programs that expand financial inclusion, particularly investments in accessible and appropriate financial products, should be developed to reach and cover the financially underserved, especially those with limited financial literacy and restricted access to mainstream financial services.

